# Construction of an Anthropomorphic Phantom for Use in Evaluating Pediatric Airway Digital Tomosynthesis Protocols

**DOI:** 10.1155/2018/3835810

**Published:** 2018-04-18

**Authors:** Nima Kasraie, Amie Robinson, Sherwin Chan

**Affiliations:** Department of Radiology, Children's Mercy Hospital, 2401 Gillham Rd., Kansas City, MO 64108, USA

## Abstract

Interpretation of radiolucent foreign bodies (FBs) is a common task charged to pediatric radiologists. The use of a motion compensated technique to decrease breathing motion on images would greatly decrease overall exposure to ionizing radiation and increase access to treatment yielding a great impact on clinical care. This study reports on the methodology and materials used to construct an in-house anthropomorphic phantom for investigating image quality in digital tomosynthesis protocols for volumetric imaging of the pediatric airway. Availability and cost of possible substitute materials were considered and simplifying assumptions were made. Two different modular phantoms were assembled in coronal slab layers using materials designed to approximate a one- and three-year-old thorax at diagnostic photon energies for use with digital tomosynthesis protocols such as those offered on GE's VolumeRAD application. Exposures were made using both phantoms with inserted food particles inside an oscillating airway. The goal of the phantom is to help evaluate (1) whether the currently used protocol is sufficient to image the airway despite breathing motion and (2) whether it is not, to find the optimal protocol by testing various commercially available protocols using this phantom. The affordable construction of the pediatric sized phantom aimed at optimizing GE's VolumeRAD protocol for airway foreign body imaging is demonstrated in this study which can be used to test VolumeRAD's ability to image the airways with and without a low-density foreign body within the airways.

## 1. Introduction

Accidental impaction of objects in pediatric respiratory tract, known as airway foreign bodies (AFBs), is a common and potentially life-threatening occurrence. Of the 110,000 foreign body ingestions in patients of all ages reported in the United States in 2011, over 85% of these occurred in the pediatric population [1], and this continues to remain the most common cause of mortality owing to unintentional injury in children aged under 1 year [2].

Aspiration of a foreign body can be difficult to diagnose especially in infants and small children as most aspirated objects are radiolucent and are not seen on routine chest X-rays [3]. Thus, conventional radiographs used in the diagnosis have low specificity for radiolucent foreign bodies [3–7]. On the other hand, digital tomosynthesis (DT) volumetric imaging has multiple clinical applications for adults, including airway imaging [8–10]. However, corresponding pediatric applications have yet to be developed, as pediatric imaging presents a unique set of challenges for DT volumetric acquisition, the largest of these being the challenge of patient cooperation during the exam. In particular, midexposure patient motion remains a resilient obstacle facing tomosynthesis imaging of the thoracic region. This is mainly due to the long time spans (greater than ten seconds) typically used by thoracic protocol exposures to complete their imaging sweep. This motion can be classified into respiratory and patient body motions (e.g., child wiggling and unrest). In pediatric imaging, radiologists are able to compensate for the latter using various immobilization devices for younger aged children as well as child life specialists encouraging patient cooperation. Nonetheless, the majority of pediatric patients will not be able to exercise full control over their breathing during exams, especially if they are acutely symptomatic (e.g., shortness of breath, coughing, or choking).

Quantifying respiratory patient motion may be very useful in assessing which pediatric clinical applications would be appropriate targets for tomosynthesis imaging. In this study, we aim to investigate the proposal to evaluate the amount of respiratory motion in the pediatric airway. Computational modeling studies have shown that DT may add substantial sensitivity and specificity for the detection of low-density aspirated foreign bodies; thus, we believe that airway tomosynthesis would be a highly useful tool for pediatric radiologists. Our preliminary investigation has shown that adding simulated VolumeRAD images to simulated radiographs increased sensitivity from 15% to 67% and increased specificity from 94% to 100% [11].

Interpretation of radiolucent foreign bodies (FBs) is a common task charged to pediatric radiologists. The use of a motion compensated technique to decrease breathing motion on images would greatly decrease exposure to ionizing radiation and increase access to treatment yielding a great impact on clinical care. One of the main disadvantages of tomosynthesis is the long acquisition time which makes it very susceptible to motion degradation of image quality. By improving image quality, we could improve diagnostic performance and tomosynthesis could replace CT as a confirmatory test in some cases. If tomosynthesis is used to replace chest CT for any clinical indication [12], then the dose saving could be considerable since the effective dose from CT for an adult patient is in the range of 4.0–18.0 mSv [13]. Our research group is interested in testing the hypothesis that patient breathing motion will not degrade VolumeRAD image quality enough to significantly affect the ability of radiologists to diagnose a low-density foreign body in the airway. The primary aim of this paper is to describe the construction of a phantom that mimics the breathing motion of infants and small children who are prone to ingesting objects in their airways. The phantom is to be used to test different VolumeRAD protocols to determine which one is optimally suited to minimize breathing artifacts and create the images that are best fit for diagnosis of pediatric AFBs. This report focuses on the design, construction, and feasibility testing of this phantom for the aforementioned purpose.

## 2. Methods and Materials

First, the tissue-equivalent substitutes used were developed with three design benchmarks in mind: approximating the physical properties of human tissue such as density, attenuation coefficients, and physical dimensions. In regard to the latter design element, the phantom was designed to mimic the thorax habitus of a one- and a three-year-old patient. The selected age is based on the fact that this age group in our practice is known to exhibit a higher occurrence of aspirating a foreign body, and published reports show that 80% of all AFB cases occur below the three-year-old age group [14].

Second is compartmentalizing the phantom into modular segments to enable switching out various elements such as airways of different sizes or to improve or add components if need be, without the need to change the entire phantom set for each modification.

The third design element focused on particular geometry of the tomosynthesis acquisition. Unlike the conventional oval-shaped chest dosimetry or computed tomography (CT) phantoms, the cranial-caudal (CC) direction of the sweep movement during the sequence of projections in VolumeRAD eases restrictions on having to consider an oval-shaped architecture in the transverse axial plane for this phantom prototype. Thus, the vertical single-plane movement of the sweep allows the use of a simplified Cartesian model design for the shape of the phantom. To accommodate the above stated goals, we employed the materials described in the following sections in constructing the phantom.

The developed lung and tissue-equivalent materials were evaluated by measuring the attenuation properties, namely, the Hounsfield Unit (HU) values for each component, using a Siemens Somatom Flash 64-slice CT scanner operated at a tube voltage of 120 kVp and employing a mA modulated exposure control. The mean HU was determined from three selected regions of interest (ROI) at different axial (*z*) positions in the phantom, using areas of approximately 100 mm^2^.

Density measurements of each sample were taken utilizing Archimedes' principle. A cured sample of each material was weighed on an APX-60 model scale with 0.1 mg precision (Denver Instrument, Bohemia, NY) to find the dry mass of each sample. The samples were then submerged in a beaker of deionized water to estimate the volume of the samples.

The most common site for AFBs is the right lower bronchus or its bronchus intermedius [15]. The positioning of the lodged food particles in our phantom involved the right and left bronchi with equal frequency and was based on pediatric data cited by Rothmann and Boeckman [16]. We used dry food particles, namely, peanuts (the most common food type), accounting for 35%–55% of all aspirated foreign bodies, as well as seeds, popcorn, and other food particles [16].

### 2.1. Phantom Construction and Imaging Methodology

The design of the phantom consists of three slabs stacked together: a posterior slab, a midsection slab, and an anterior slab. The construction is modular: any slab can be removed or swapped so as to change the configuration (AP length or components) of the phantom if desired.

Two phantom prototypes were made, one with a larger AP dimension and slightly different anterior lung design. The respective thickness (or AP length) of each slab of the first phantom is 80 mm for the anterior section, 22 mm for the middle slab, and 58 mm for the posterior part combining to a sum of 160 mm, roughly corresponding to the chest of an average three-year-old, while the respective thickness (or AP length) of each slab of the second phantom is 52 mm for the anterior section, 22 mm for the middle slab, and 58 mm for the posterior part combining to a sum of 132 mm, roughly corresponding to the chest of an average one-year-old child. The only differences between the two phantom prototypes essentially were the anterior slab AP length and middle slab airway size.

The overall AP thicknesses we used were based on the measurements made by Kleinman et al. where we tried to select an AP thickness above the 50th but below the 95th percentile of AP thorax values for one- and three-year-olds, respectively [17]. The linear equation of the 50th percentile of their data (see (1)) can roughly be used to scale this thickness to other thorax sizes (including adult) if need be. Here, *y* is the AP dimensions in centimeters and *x* is age in years:

(1) $$\begin{array}{c} y=0.60x+11.7. \end{array}$$

The middle slab is filled with water to enable movement of the airways, which is why we decided to use VolumeRAD's vertical or wall-stand acquisition protocol. The other two sections are constructed as box-shaped frames with bone-tissue-equivalent (BE) inserts glued to the anterior rim of the interior of the container at anatomical spacing and then poured and filled with the soft-tissue equivalent (SE) epoxy resin.

**Figure 1:**
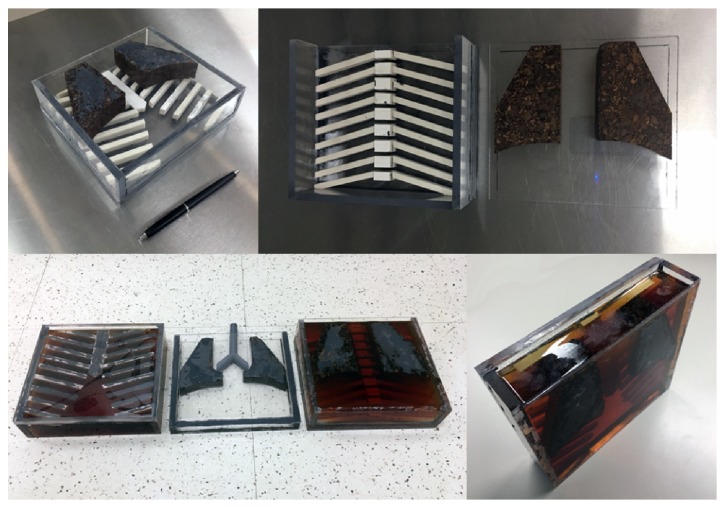
Components of the phantom assembly. Clockwise from top left: the anterior frame with LE and BE inserts. The posterior frame with the BE insert and the LE insert not attached yet. The anterior slab view from the side with the SE poured in. All 3 slabs side by side for comparison with the SE poured in A and P frames.

The frame or container itself is made of cast acrylic (Regal Plastics, Kansas City) with dimensions of 20 cm in the transverse and 18 cm in the CC (or vertical) direction for all three slabs (Figure 1) in both phantom types.

All exposures of the phantom were acquired with a GE Discovery XR 650 unit using the “Chest VolumeRAD” protocol. The tomosynthesis angle was 30 degrees and the acquisition time was 11.4 seconds.

### 2.2. Soft-Tissue Equivalent (SE) Substitute

Adipose tissue was not specifically modeled in the construction of this anthropomorphic phantom. The distribution of subcutaneous as well as intra-abdominal adipose tissue was determined to be too complicated to directly model with a specific tissue-equivalent material. Thus, the SE substitute was developed to be a homogeneous soft-tissue analog that represents skeletal muscle as well as organs, connective tissue, and adipose tissue.

A polyurethane-based SE substitute was used to match the X-ray attenuation and density of human soft tissue within the diagnostic (80–120 kVp) energy range. Polyurethane-based material has been used for constructing lung phantoms in the past [18]. Hence, the SE substitute was designed to have a density similar to that of human soft tissue (1.04 g/cm^3^) and with published [19, 20] X-ray mass attenuation coefficients of soft-tissue compositions in mind.

The commercially available, two-part rubber compound PMC 121/30 Wet (Smooth-On Inc., Easton, PA) was used as the template for soft-tissue equivalent inserts. The durable, readily available, polyurethane-based compound was relatively easy to work with at room temperature; however, a hood or sufficient ventilation is required to ensure respiratory protection of the user and nearby occupants. Part A of the compound was a TDI prepolymer composed of diisononyl phthalate and toluene diisocyanate. Part B was composed of diisononyl phthalate, diethyltoluenediamine, and phenylmercury neodecanoate. The two parts were thoroughly mixed in 1 : 1 volume (or mass) ratios in a disposable graduated cylinder, as per instructions by the manufacturer. We ended up requiring nearly 2000 ml of the mixed compound, per phantom set. The pouring had to be planned ahead of time and the phantom frame constructed beforehand, because once mixed, the composite liquid exhibits high viscosity (1800 CPS, per technical specs) which makes handling rather difficult and has a pot life of less than 30 minutes, with the viscosity gradually increasing by the minute. The pouring has to be slow to avoid trapping air bubbles in the phantom. This is a very crucial factor to ensure a homogenous mix. The poured mixture was given 24 hours of curing time, and four to eight hours of postcuring heating inside an oven to allow the resin to settle in and permanently solidify. Our oven (Mac Medical, Millstadt, IL) was set at 130 F overnight. Our design required no release agent as our phantom frame served as the casting mold to the mixture. The manufacturer's technical overview sheet claims a specific gravity of 1.04 g/cm^3^ for this material.

### 2.3. Lung-Tissue Equivalent (LE) Substitute

**Figure 2:**
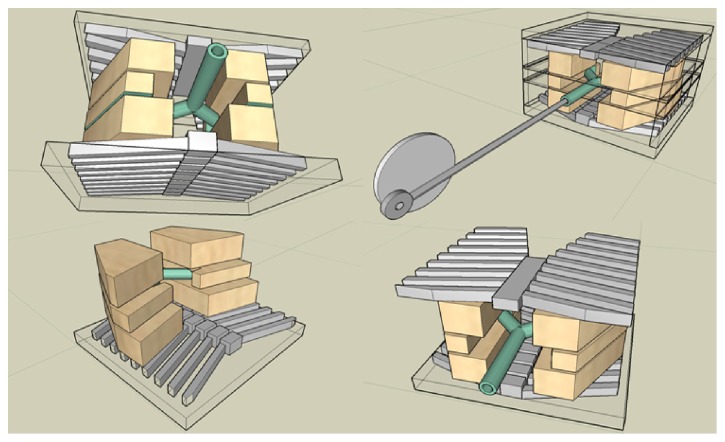
Rendering of phantom design and components. 3D perspective view of renderings of the second phantom, using FORMZ CAD software, showing different parts of the phantom without the SE. Notice the gradual stepped contour of the inferior part of the 6-plated lung in the lower left rendering.

The LE substitute used was designed by combining commercially available dark cork tiles measuring 30.4 × 30.4 × 1.0 cm (ArtMinds, Michaels, Irving, TX) and cutting them into coronal plates in four different quadrants (anterior left, anterior right, posterior left, and posterior right) and gluing them together using a two-part epoxy mix adhesive (Quiksteel Blue Magic, Cleburne, TX) into stacks of 3.0 and 6.0 cm anterior, 3.0 and 4.0 cm posterior, and 1.5 cm midsection thicknesses. The superior lobes were cut to measure 3.0 cm in the transverse direction and widened as we move toward the inferior lobes. When cutting, each of the six plates had a slightly different contour in the bottom (inferior) section, so that when the stacks are aligned, the overall shape of the lung changed stepwise going from anterior to posterior, in line with the sloped shape of the costodiaphragmatic recess area of the lungs (seen in Figure 2). The right lung was also intentionally slightly elevated compared to the left one, representing normal anatomical configuration. The cork material was selected by trial and error among other candidates due to its amorphous texture, light density, attenuation properties, and ease of handling and reproducibility. While the density of lung tissue can vary widely depending on the level of inspiration, patients undergoing diagnostic procedures are typically asked to hold their breath during the exposure. Therefore, a value of 0.33 g/cm^3^ was chosen for the LE substitute, representing the density of a fully inspired lung [19].

### 2.4. Bone-Tissue-Equivalent (BE) Substitute

**Figure 3:**
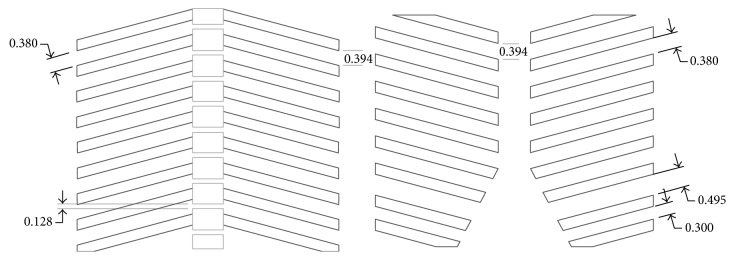
Spacing of BE components in inches. Dimensions of the anterior and posterior rib cage used in the second version of the phantom.

The BE substitute used was a Gammex 450-210 cortical bone-tissue-equivalent material (Gammex Inc., Middleton, WI) used for dosimetry studies. A 20 × 20 × 1.0 cm plate of this material was cut using a precision water jet technique (Kastle Grinding, Lee's Summit, MO) and the input computer-aided design (CAD) spacing dimensions seen in Figure 3. The absorption and scattering properties of this material “are within one percent of living tissue” and provide adequate simulations for electron and photon applications between 0.01 and 100 MeV, according to the manufacturer [21]. The dimensions of the cut pieces were as follows: rib thickness, 0.7 cm; rib spacing, 1 cm; sternum, 0.6 cm AP × 2 cm transverse × 9 cm in CC direction; spine, 2 cm transverse × 2 cm AP, spanning the full length of phantom in the CC direction. These dimensions (see Figure 3) were based on a normal CT from a three-year-old patient at our institution.

### 2.5. The Airway Equivalent (AE) Substitute

**Figure 4:**
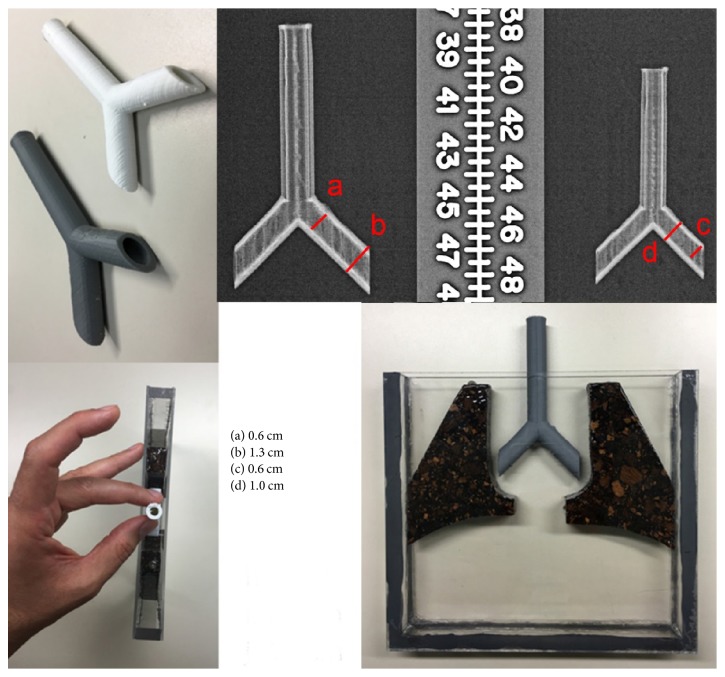
View of the AE components and how they fit within the middle slab of the phantom. Clockwise from top left: multiple copies of two sizes were printed. The linear dimensions are given for the X-ray image with a centimeter scale appearing for comparison. The angle at the carina between the right and the left mainstem bronchi is 90 degrees. The tubes are continuously hollow and sealed airtight before being inserted and imaged as part of the phantom. A side view showing how the airway fits in the middle slab. A 1 mm gap between the LE component and the container (lower left image) allows for water to uniformly fill any space not occupied by the LE or AE substitutes. An AP view.

The main components of an airway (i.e., trachea, carina, left mainstem bronchus, and right mainstem bronchus) were all made using onsite 3D printers using Platinum Series ABS Filaments (Airwolf 3D Printers, Costa Mesa, CA) which are composed of acrylonitrile-butadiene-styrene copolymer. This material has specific gravity (density) of 1.03–1.10 g/cm^3^ and is insoluble in water. Using two Airwolf HDX model units 150526-002 and 150617-0001 (Airwolf 3D Printers, Costa Mesa, CA), we printed 20 airways in two different sizes, as shown in Figure 4: ten airways for a one-year-old and ten larger airway sizes corresponding to a three-year-old. The 3D dataset used to print the models was derived from two normal chest CTs of a one- and a three-year-old who were scanned at our institution. The inner diameter of the printed trachea of the one-year-old was 6 mm and the outer was 10 mm, while the lumen (inner) diameter of the printed trachea in the three-year-old was 6 mm and the outer diameter was 13 mm (Figure 4). The tubes are continuously hollow and airtight when sealed at the ends. The top of the tubes is attached to a shaft that connects to the rotor that oscillates the airway to breathing frequencies of 30 cycles/min (for 1 yr old) and 20 cycles/min (for 3 yr old) [22, 23]. Twenty copies were printed so as to test different types and locations of foreign bodies inserted into the lumen before being sealed and inserted into the phantom. In order for the airway tube to be able to freely move with the breathing frequency, it was necessary to carve out a groove in the medial part of the LE in the middle slab of the phantom, as seen in the lower right image in Figure 4. The remaining space in the middle slab was filled with water to mimic the soft tissues of the mediastinum that surround the airway.

### 2.6. Simulated Motion

We used a rotating motor with an adjustable frequency and attached the 3D-printed airway to the motor using a rigid rod. As the motor would rotate at a desired frequency based on the estimated heart rates of pediatric patients, the rotational motion would translate into vertical motion of the printed airway. This vertical motion simulated the vertical motion of the airway in the chest caused by diaphragm movement.

## 3. Results and Discussion

To date, two pediatric thorax phantoms, one mimicking a one-year-old's chest and one mimicking a three-year-old's chest, have been constructed using the methods and materials described in the previous section. The major difference between these versions is that the first model was thicker in the AP dimension than the second with different airway sizes.  Table 1 shows the average of the measured CT numbers (in HU) of the phantom versus a sample three-year-old patient for the four key components.  Table 2 shows the half value layer (HVL) thicknesses (in mm) evaluating the beam itself and the phantom at 60, 80, 100, and 120 kVp using a chest technique with 2 mAs.  Table 3 displays the measured density of each component compared to some values in the referenced literature [24, 25].

**Figure 5:**
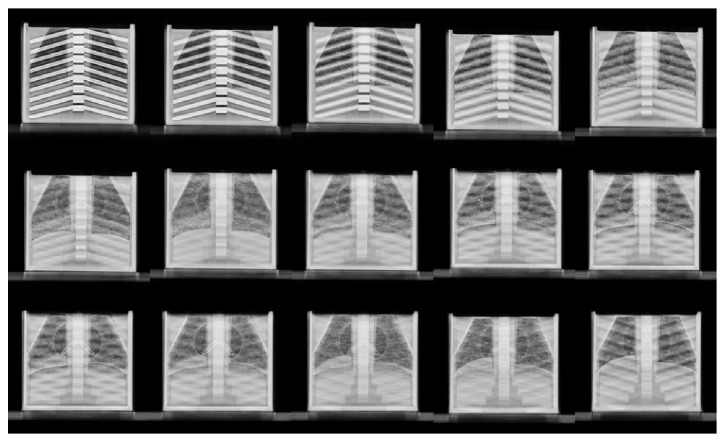
A montage of 12 reconstructed frames of the VolumeRAD acquisition for the first phantom. Notice how the airway bronchi are clearly visible in frames 10 through 12 (left to right, top to bottom).

**Figure 6:**
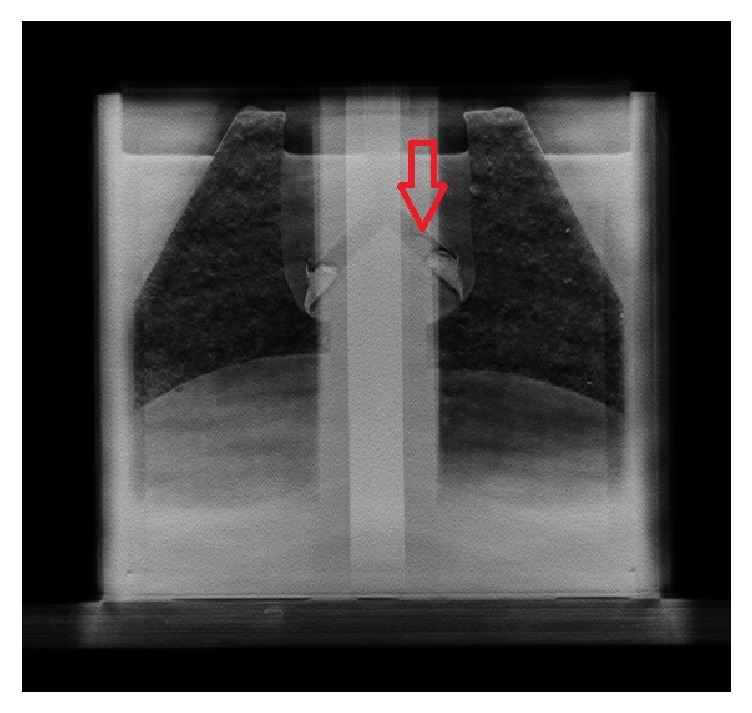
Visualization of a food particle in the airway. A reconstructed frame of the VolumeRAD acquisition for the second phantom with a low-density dry food particle lodged in the right airway bronchus (red arrow).

Figure 5 shows a montage of select frames from the reconstructed images for phantom one, while Figure 6 shows one of those corresponding frames from the second phantom with a food particle lodged in one of the bronchi.

A series of 80 simulated images were interpreted for the presence of a radiolucent AFB by a designated pediatric radiologist. 40 simulated images were chest X-rays only and 40 images were DTS images. Twenty images in each group were static and 20 were motion-simulated to represent breathing. Seventeen (ten in motion group and seven in static group) were scored as uninterpretable by the reader and were excluded from our final analysis. Scoring was based on a 5-point probability Likert scale: (1) not probable, (2) somewhat improbable, (3) neutral, (4) somewhat probable, or (5) very probable for right bronchus, mainstem bronchus, and left bronchus. Images were viewed on our institutional PACS system and compared to ground truth.

After removal of the seventeen images, we compared the two groups to the ground truth. Overall, in comparison to the ground truth, the reader correctly identified the presence or absence of a foreign body in 44% (*n* = 28/63) of the images. In comparison to the ground truth and static versus motion images, the static images were correctly identified in 48% (*n* = 16/33) of the cases and in 40% (*n* = 12/30) of the motion group.

Winslow et al. enumerate several advantages of using a polyurethane-based material for constructing a dosimetry phantom [25]. The same reasoning applies to the phantom design in this study whose purpose is diagnostic in nature. However, there is room for improvements to be made in the material design and selection. For example, the only material available to us for the 3D printer was the commercial spool of acrylonitrile-butadiene-styrene copolymer threads. A better material can be substituted, which would improve the density and attenuation coefficient values to closer physiological values as those depicted in Tables 1 and 2. Similarly, among the limitations of the phantom also was the not too large but finite difference in CT numbers between the phantom (9.8 HU) and anatomical range (45.6 HU) for the SE material. This problem can theoretically however be mitigated by adding traces of impurities to the SE mixture before curing. For example, by adding small quantities of hydroxyapatite (number 289396) in powder form (Sigma-Aldrich, St. Louis, MO) to the polyurethane PMC 121/30 liquid form mixture, it is possible to elevate the attenuation of the SE material to higher values, provided that the compound is thoroughly mixed while parts A and B of the mixture are being poured into the phantom template. Likewise, low-density esters or fatty liquids can also be added to the curing mixture to lower the CT numbers closer to desired values. This CT number matching problem was particularly pronounced with the BE (measured 1111.1 HU versus actual 346.9 HU) material. The latter however also can be addressed by noting that a different model of the Gammex tissue-equivalent slab, namely, “inner bone” Model 456 instead of cortical bone (Model 450), which has a much lower attenuation for the area of interest in the study, can be used to construct the BE substitute inserts. An improvement in the DT image quality would be expected using a lesser attenuating bone material.

If a horizontal (instead of vertical) orientation of the setup is desired, a solid tissue-equivalent material can be used in place of water for the midsection slab (e.g., Gammex 452 Muscle or a tissue mimicking gel). The entire phantom can then also be used for hypothetical horizontal sweeps. We however only performed the imaging in vertical mode to be clinically relevant.

Finally, in our practice, we currently use a 2-view plus bilateral decubitus radiographic exam for pediatric foreign body airway imaging. However, standard radiographs with or without special views (decubitus or expiratory) suffer from low diagnostic accuracy [6]. We did not perform any dosimetry measurements in the present study and instead only focused on the visualization aspect of the AFB imaging. However, comparative dosimetry studies have been performed on DT acquisitions [26, 27]. For example, Bath et al. reported the effective dose to a standard-sized patient from a VolumeRAD chest tomosynthesis examination to be close to 2% of an average chest CT and only two to three times the effective dose from the conventional (standard only) two-view chest radiography examination [28].

The purpose of this project was to construct a phantom for use in observer studies to measure the change in diagnostic accuracy for detecting low-density AFB due to breathing motion using a DT radiographic technique. This is the first and only phantom of its kind the authors of this paper are aware of specifically designed for a pediatric target.

## 4. Conclusions

This study reports a methodology developed to construct anthropomorphic phantoms for use in radiographic tomosynthesis studies. While the value of this methodology has already been proven with the construction of two pediatric phantoms, it should be noted that the same methodology could be applied to the construction of phantoms of other sizes and ages. In particular, our group plans to develop and use these phantoms for dynamic studies in order to accurately model the moving airways. Furthermore, such phantoms may aid optimization studies regarding kVp, number of projections, total angular range, and geometric acquisition parameters that affect DT image quality and dosimetry [27].

While anthropomorphic phantoms have many potential applications, this particular phantom series was created to evaluate tomosynthesis techniques in radiographic units for the purpose of visualizing low-density foreign body particles in pediatric airways. In our small study, we used this phantom that showed that diagnostic accuracy was better in static images compared to images with simulated breathing motion. This result contradicted our main hypothesis that breathing motion would not affect diagnostic accuracy of digital tomosynthesis. More studies are needed to confirm these results and this phantom would be an ideal tool with which to do those studies. It is anticipated that other institutions could create similar customized phantoms for clinical use by following the methodology in this paper and using the described tissue-equivalent materials for a total material cost of less than US$ 1,000.

## Figures and Tables

**Table 1:** Measured (average/standard deviation) attenuation values in HU of key phantom components vs. an actual patient with same ROI (100 mm^2^).

	SE	BE	LE	AE (wall)	Acrylic
phantom	9.8/11.0	1111.1/130.9	−664.6/84.8	−122.9/7.3	121.2/9.5
actual	45.6/7.9	346.9/73.5	−652.8/78.9	62/80	NA

**Table 2:** Measured Half Value Layer (HVL) thicknesses of the phantom (in mm) at 60, 80, 100, and 120 kVp energy for the X-ray unit used.

	60	80	100	120
Plain beam (mm Al)	2.4	3.2	3.9	4.7
Phantom (mm)	3.4	4.2	4.8	5.5

**Table 3:** Measured average density values of key phantom components vs. actual reported values from literature (g/cm^3^).

	SE	BE	LE	AE (wall)	Acrylic
phantom	0.97	1.89	0.38	0.89	1.23
actual	1.04	1.85	0.33	1.1	
